# In Vivo Classification and Characterization of Carotid Atherosclerotic Lesions with Integrated ^18^F-FDG PET/MRI

**DOI:** 10.3390/diagnostics14101006

**Published:** 2024-05-13

**Authors:** Fan Yu, Yue Zhang, Heyu Sun, Xiaoran Li, Yi Shan, Chong Zheng, Bixiao Cui, Jing Li, Yang Yang, Bin Yang, Yan Ma, Yabing Wang, Liqun Jiao, Xiang Li, Jie Lu

**Affiliations:** 1 Department of Radiology and Nuclear Medicine, Xuanwu Hospital, Capital Medical University, Changchun Street, No. 45, Beijing 100053, China; yufan131027@163.com (F.Y.); markweal_image@163.com (Y.Z.); heyusun2024@126.com (H.S.); xiaoranli2024@sina.com (X.L.); shanyiedu@hotmail.com (Y.S.); 17812089921@163.com (C.Z.); bixiao1311@163.com (B.C.); bhlijing13@163.com (J.L.); 2 Beijing Key Laboratory of Magnetic Resonance Imaging and Brain Informatics, Beijing 100053, China; 3 Beijing United Imaging Research Institute of Intelligent Imaging, Beijing 100094, China; yang.yang03@cri-united-imaging.com; 4 Department of Neurosurgery, Xuanwu Hospital, Capital Medical University, Changchun Street, No. 45, Beijing 100053, China; yangbin_81@163.com (B.Y.); leavesyan@sina.com (Y.M.); wangyabing@foxmail.com (Y.W.); liqunjiao@sina.cn (L.J.); 5 China International Neuroscience Institute (China-INI), Beijing 100053, China; 6 Department of Interventional Neuroradiology, Xuanwu Hospital, Capital Medical University, Changchun Street, No. 45, Beijing 100053, China; 7 Division of Nuclear Medicine, Department of Biomedical Imaging and Image-Guided Therapy, Vienna General Hospital, Medical University of Vienna, 1090 Vienna, Austria; 8 Department of Nuclear Medicine, Beijing Chest Hospital, Capital Medical University, Beijing Tuberculosis and Thoracic Tumor Research Institute, Beijing 101149, China

**Keywords:** ^18^F-FDG, atherosclerosis, carotid arteries, MRI, PET, stroke, vulnerable plaque

## Abstract

Background: The aim of this study was to exploit integrated PET/MRI to simultaneously evaluate the morphological, component, and metabolic features of advanced atherosclerotic plaques and explore their incremental value. Methods: In this observational prospective cohort study, patients with advanced plaque in the carotid artery underwent ^18^F-FDG PET/MRI. Plaque morphological features were measured, and plaque component features were determined via MRI according to AHA lesion-types. Maximum standardized uptake values (SUV_max_) and tissue to background ratio (TBR) on PET were calculated. Area under the receiver-operating characteristic curve (AUC) and net reclassification improvement (NRI) were used to compare the incremental contribution of FDG uptake when added to AHA lesion-types for symptomatic plaque classification. Results: A total of 280 patients with advanced plaque in the carotid artery were recruited. A total of 402 plaques were confirmed, and 87 of 402 (21.6%) were symptomatic plaques. ^18^F-FDG PET/MRI was performed a mean of 38 days (range 1–90) after the symptom. Increased stenosis degree (61.5% vs. 50.0%, *p* < 0.001) and TBR (2.96 vs. 2.32, *p* < 0.001) were observed in symptomatic plaques compared with asymptomatic plaques. The performance of the combined model (AHA lesion type VI + stenosis degree + TBR) for predicting symptomatic plaques was the best among all models (AUC = 0.789). The improvement of the combined model (AHA lesion type VII + stenosis degree + TBR) over AHA lesion type VII model for predicting symptomatic plaques was the highest (AUC = 0.757/0.454, combined model/AHA lesion type VII model), and the NRI was 50.7%. Conclusions: Integrated PET/MRI could simultaneously evaluate the morphological component and inflammation features of advanced atherosclerotic plaques and provide supplementary optimization information over AHA lesion-types for identifying vulnerable plaques in atherosclerosis subjects to achieve further stratification of stroke risk.

## 1. Introduction

Atherosclerotic carotid artery disease is found in 15–20% of patients who present with ischemic stroke or transient ischemic attack [1]. The global prevalence of atherosclerotic carotid plaque is 21.1%, equivalent to 815.76 million affected people [2]. The most important mechanism by which carotid plaque causes stroke or transient ischemic attack is plaque rupture [3]. Such carotid plaques, with a high risk of rupture, are so-called vulnerable plaques. 

Degree of luminal stenosis measured by ultrasound serves as the sole morphological imaging marker for selecting vulnerable plaque to undergo a therapeutic approach. However, several trials found discrepancy in absolute risk reduction in patients with the same degree of luminal stenosis, highlighting the importance of factors other than degree of luminal obstruction in determining risk [4,5,6]. Thanks to developments in high-resolution MRI, several component biomarkers, such as intraplaque hemorrhage, lipid core, and irregular plaque surface have emerged in characterizing the vulnerability status of plaques [7,8,9]. Further, a scoring system (American Heart Association [AHA] lesion-types) based on plaque component was proposed to assess plaque vulnerability. However, even with the help of machine learning, the diagnostic test based on single imaging biomarker mentioned above showed only average validity [10], highlighting the demand for advanced evaluation.

Recent advances in basic science have established the fundamental role of inflammation in all stages of atherosclerosis, shifting the focus to inflammation evaluation [11]. ^18^F-FDG is the most widely used radiotracer for the molecular imaging of atherosclerosis. Currently, FDG PET/CT is providing new insights on metabolic evaluation-based stroke risk classification [12]. The incremental value of PET over CT was demonstrated in a longitudinal study; however, the component evaluation was neglected [13]. Integrated PET/MRI could measure both PET activity and plaque component at the same time. However, the study was focused only on the prevalence of coincident FDG uptake in plaques detected by MRI [14]. The incremental value of PET over MRI in carotid plaque evaluation is still unclear.

In the current study, we aim to exploited integrated PET/MRI to simultaneously evaluate the morphological component and metabolic features of advanced atherosclerotic plaques and explore the incremental value of PET/MRI.

## 2. Materials and Methods

### 2.1. Patient Selection

Subjects were recruited for this observational, prospective cohort study at the National Center for Neurological Disorders between September 2020 and December 2022. Subjects were offered a carotid ^18^F-FDG PET/MRI study if they met the following clinical criteria: (a) aged 45–85 years, (b) presence of advanced plaque in unilateral or bilateral carotid artery determined by ultrasonography (wall thickening >1.5 mm [15]), and (c) no contraindications for MR imaging or contrast material injection. Exclusion criteria were: (a) prior carotid endarterectomy, carotid stenting, or neck radiation therapy, (b) any prior cancer or chemotherapy history, (c) presence of acute or chronic inflammatory or autoimmune disease (based on documented medical history) or use of chronic anti-inflammatory therapy at the time of PET/MR imaging, (d) poor image quality. Demographics and clinical information were recorded. This study was approved by Xuanwu Hospital Medical Ethics Committee ([2022]023). All participants provided written informed consent.

### 2.2. Acquisition Protocols with the Integrated PET/MRI System

PET and MR vessel wall imaging were performed simultaneously. All patients were asked to fast for at least 6 h before ^18^F-FDG PET imaging. Imaging was performed only if fasting glucose was lower than 7.7 mmol/L before tracer injection. ^18^F-FDG was injected intravenously at a dose of 3–4 MBq/kg. The acquisition started 90–120 min after tracer injection using an integrated PET/MRI system (uPMR790, United Imaging Healthcare, Shanghai, China). MR vessel wall imaging was performed with an 8-channel carotid coil. The PET/MR image acquisition range was centered on the carotid bifurcation, with an 18 cm coverage. High-resolution carotid vessel wall MRI sequences included 3D TOF-MRA, 3D T1, 3D T2, and post-contrast (gadolinium-DTPA, 0.1 mmol/kg, 2.5 mL/s) enhanced 3D T1 (T1C+). The detailed MRI acquisition parameters are listed in Supplemental Table S1. PET images were reconstructed using a hyperiterative algorithm (matrix size = 256 × 256, and thickness = 1.4 mm) with time-of-flight information following data corrections for attenuation, scatter, and random coincidences.

### 2.3. Analysis of FDG PET/MR Images

PET/MR image clinical conventional analysis was performed using a dedicated plaque analysis workstation (United Imaging Healthcare, Shanghai, China). MR images were analyzed by three experienced radiologists (YZ, CZ, and YS; >10 years of experience in neuroradiology) who were blinded to all other information, of which two (YZ and CZ) independently reviewed the images. If the review results were inconsistent, a third radiologist (YS) performed a peer review of the inconsistent MR images. For clinical qualitative analysis, plaque composition and surface status were classified according to AHA lesion-types [16]: (a) Type I–II: near-normal wall thickness, (b) Type III: diffuse intimal thickening or small eccentric plaque with no calcificationno calcification, (c) Type IV–V: plaque with a lipid or necrotic core surrounded by fibrous tissue with possible calcification, (d) Type VI: complex plaque with possible surface defect, hemorrhage, or thrombus, (e) Type VII: calcified plaque, or (f) Type VIII: fibrotic plaque without lipid core and with possible small calcifications. For quantitative morphological analysis, the degree of stenosis (NASCET), plaque area, and remodeling index were automatically calculated at the most stenotic slice by manually drawing regions of interest. The SUV_max_ of the plaque was calculated as the maximum of all plaque slices by delineating the circular regions of interest of each slice. The maximum target-to-background ratio (TBR_max_) was calculated as the ratio of SUV_max_ to the venous blood pool SUV_mean_ [17,18].

### 2.4. Clinical Evaluation

Clinical evaluation was performed by two experienced neurosurgeons (10 years’ experience in neurovascular surgery, BY, YBW). An atherosclerotic carotid plaque was considered a symptomatic plaque when it was within the ipsilateral carotid artery territory upstream from a confirmed transient ischemic attack and ischemic stroke within 3 months [19].

### 2.5. Statistical Methods

Descriptive data are presented as mean ± standard error of the mean (SEM) for continuous parametric variables, median [interquartile range (IQR)] for continuous nonparametric data, and frequency with proportions for nominal variables as appropriate. For subgroup analysis, all plaques were divided into high and low FDG uptake groups. High FDG uptake defined as ≥median SUV_max_ of 402 advanced plaques. Independent Student’s *t* test and Mann-Whitney U test was used to compare the differences between continuous variables, and Fisher’s exact test was performed to determine the differences between categorical variables. Multivariate logistic regression analysis was performed for each single feature. We assessed the improvement in discrimination (symptomatic or asymptomatic) by comparing the area under the receiver-operating characteristic curves (AUC) in 3 models (model 1: AHA lesion type + TBR; model 2: AHA lesion type + stenosis degree; model 3: model 1 + model 2) with component alone. We assessed the classification of risk using the net reclassification improvement (NRI) formula [20]: NRI = [Prob (being correctly up-ward reclassified/event) − Prob (being incorrectly downward reclassified/event)] + [Prob (being correctly downward reclassified /nonevent) − Prob (being incorrectly classified to an upward category/nonevent)]. Statistical significance was determined if the 2-tailed probability value was <0.05. All analyses were performed using SPSS 28.0.0.

## 3. Results

### 3.1. Patient Population

A total of 280 patients (mean age 64 ± 7 years; 86.1% male) with advanced carotid plaque were included in the study. Demographic and clinical characteristics are described in Table 1. 

### 3.2. Features of Advanced Carotid Plaques on MR Vessel Wall Imaging

A total of 402 advanced carotid plaques were confirmed based on MR vessel wall imaging, and 87 of 402 (21.6%) were symptomatic plaques. ^18^F-FDG PET/MRI was performed a mean of 38 days (range 1–90) after the symptom. Quantitative analysis of morphological and component features (Table 2) found significantly higher stenosis degree (61.5% vs. 50.0%, *p* < 0.001) and higher prevalence of AHA lesion type VI (50.6% vs. 21.9%, *p* < 0.001) in the symptomatic plaques compared to asymptomatic plaques, while lower prevalence of AHA lesion type IV-V (32.2% vs. 46.3%, *p* = 0.018) and AHA lesion type VII (9.2% vs. 24.4%, *p* = 0.002) was found in the symptomatic plaques compared to asymptomatic plaques.

### 3.3. ^18^F-FDG Uptake in Advanced Carotid Plaques Measured with PET

**Figure 1:**
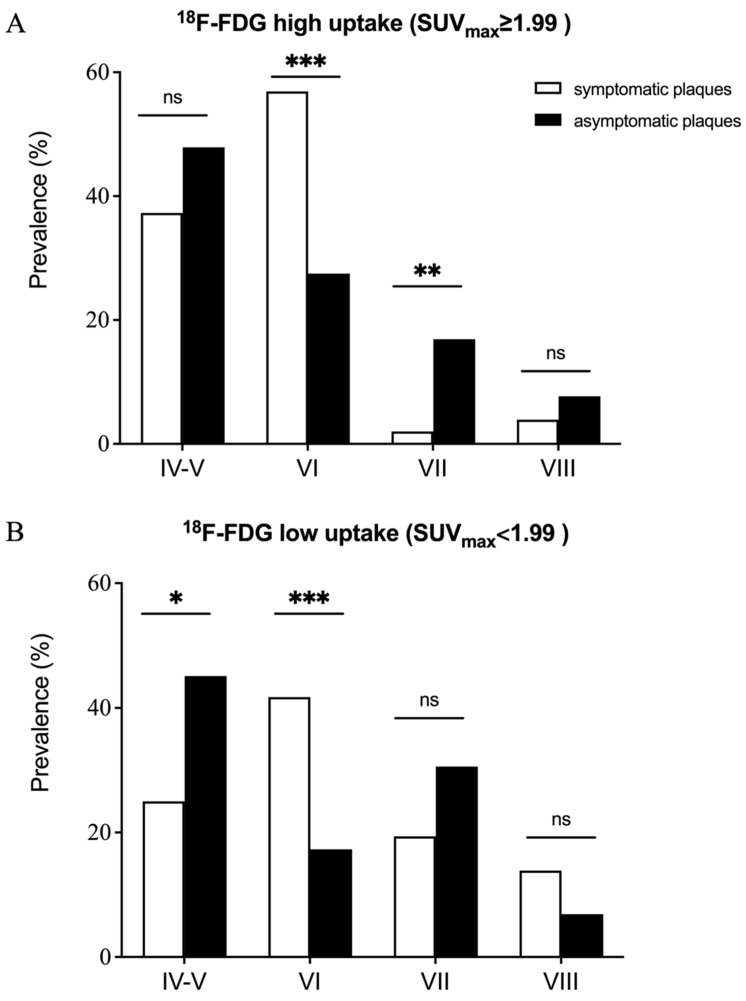
Prevalence of AHA lesion type in both the symptomatic and the asymptomatic advanced plaques in the ^18^F-FDG high uptake (**A**) and low uptake (**B**) group. Three patterns can be summarized: (1) The prevalence of AHA lesion type VI in symptomatic plaques was significantly higher than that in asymptomatic plaques for both high uptake and low uptake group. (2) No significant difference was found in the prevalence of AHA lesion type VIII between symptomatic and asymptomatic plaques for both high uptake and low uptake group. (3) The prevalence of AHA lesion type IV-V was significantly higher in the asymptomatic plaques only for low uptake group, while the prevalence of AHA lesion type VII was significantly higher in the asymptomatic plaques only for high uptake group. * *p* < 0.05, ** *p* < 0.01, *** *p* < 0.001, ns *p* > 0.05.

Increased ^18^F-FDG uptake was observed in symptomatic plaques compared with asymptomatic plaques (Table 2; SUV_max_ =2.30 (1.68–2.92) vs. 1.93 (1.52–2.49) and TBR = 2.96 (2.35–3.80) vs. 2.32 (1.81–3.00); *p* = 0.007, *p* < 0.001, respectively). Further subgroup analysis divided the advanced carotid plaques into ^18^F-FDG high uptake and low uptake group according to the median SUV_max_ (SUV_max_ ≥ 1.99 represented high uptake group). For high uptake group the prevalence of AHA lesion type VI was higher in symptomatic plaques compared with asymptomatic plaques (56.9% vs. 27.5%, *p* < 0.001, Figure 1), while the prevalence of AHA lesion type VII was lower in symptomatic plaques compared with asymptomatic plaques (2.0% vs. 16.9%, *p* = 0.006). No significant difference in the prevalence of AHA lesion type IV-V (37.3% vs. 47.9%, *p* = 0.191) and AHA lesion type VIII (3.9% vs. 7.7%, *p* = 0.350) were observed between symptomatic plaques and asymptomatic plaques. Similarly, for the low uptake group, the prevalence of AHA lesion type VI was higher in symptomatic plaques compared with asymptomatic plaques (41.7% vs. 17.3%, *p* < 0.001), and no significant difference in the prevalence of AHA lesion type VIII (13.9% vs. 6.9%, *p* = 0.292) were observed between symptomatic plaques and asymptomatic plaques. However, for the low uptake group, the prevalence of AHA lesion type IV-V was lower in symptomatic plaques compared with asymptomatic plaques (25.0% vs. 45.1%, *p* = 0.026), while no significant difference was found in the prevalence of AHA lesion type VII (19.4% vs. 30.6%, *p* = 0.177) between symptomatic plaques and asymptomatic plaques. 

### 3.4. Reclassification of Ischemic Stroke Risk

**Figure 2:**
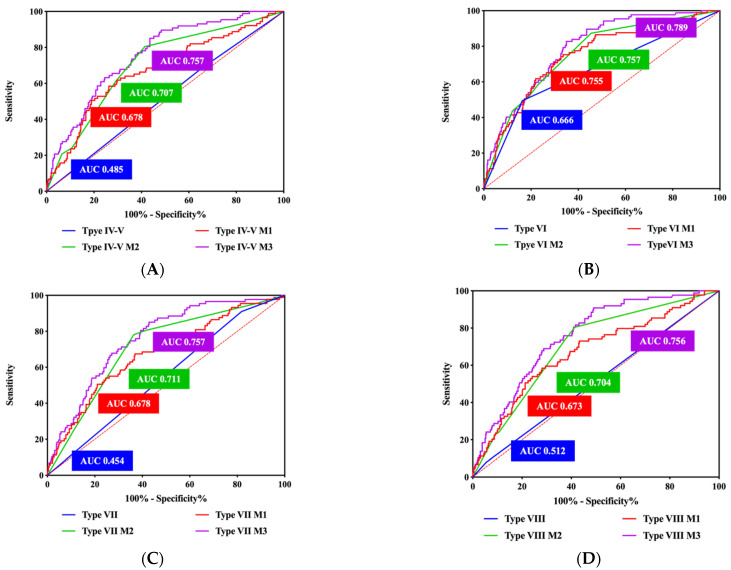
ROC of combined model in predicting symptomatic plaque. (**A**) AHA lesion type IV-V. (**B**) AHA lesion type VI. (**C**) AHA lesion type VII. (**D**) AHA lesion type VIII. ROC for AHA lesion type highlighted in blue, ROC for model 1 (AHA lesion type + TBR) highlighted in red, ROC for model 2 (AHA lesion type + stenosis degree) highlighted in green, and ROC for model 3 (AHA lesion type + stenosis degree + TBR) highlighted in purple. AUC = area under the curve; ROC = receiver-operating characteristic.

**Figure 3:**
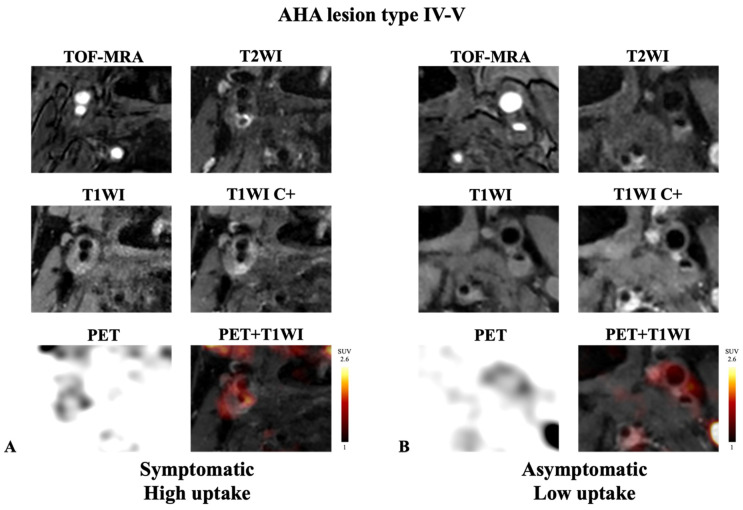
Representative example of AHA lesion type IV-V carotid plaque imaged with integrated ^18^F-FDG PET/MRI. (**A**) Symptomatic plaque with high FDG uptake. (**B**) Asymptomatic plaque with low FDG uptake. Note the presence of lipid core on post-contrast T1WI (hypo-intensity area without enhancement) in the two cases. High accumulation of ^18^F-FDG was detected with PET in the symptomatic plaque ((**A**); SUV_max_ = 2.30, TBR = 3.80). In contrast, low accumulation of ^18^F-FDG was detected with PET in the asymptomatic plaque ((**B**); SUV_max_ = 1.37, TBR = 1.37).

**Figure 4:**
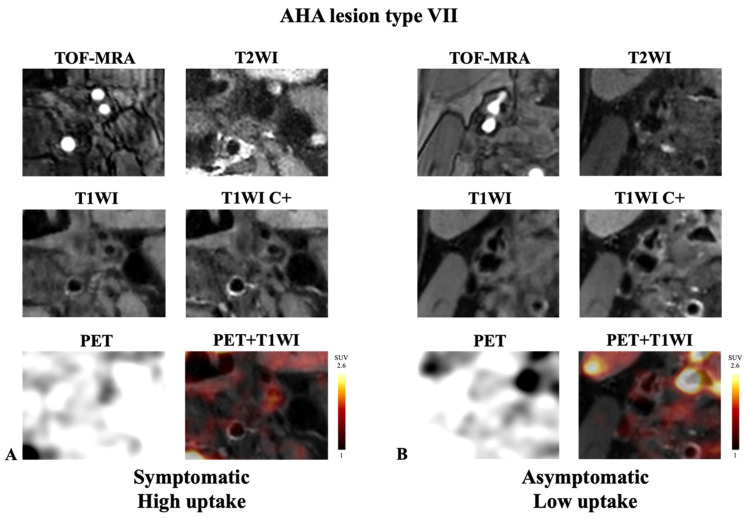
Representative example of AHA lesion type VII carotid plaque imaged with integrated ^18^F-FDG PET/MRI. (**A**) Symptomatic plaque with high FDG uptake. (**B**) Asymptomatic plaque with low FDG uptake. Note the presence of calcification on all sequences (hypo-intensity area) in the two cases. High accumulation of ^18^F-FDG was detected with PET in the asymptomatic plaque ((**A**); SUV_max_ = 3.80, TBR = 4.45). In contrast, low accumulation of ^18^F-FDG was detected with PET in the symptomatic plaque ((**B**); SUV_max_ = 1.47, TBR = 1.22).

In multivariate logistic regression analysis, stenosis degree, component features (AHA lesion type IV–V, VI and VIII) and TBR were significantly correlated with symptomatic plaques (Table 3). The AUC of stenosis degree and TBR for predicting symptomatic plaques were 0.702, 0.673, respectively. The performance of the combined model (AHA lesion type VI + stenosis degree + TBR) for predicting symptomatic plaques was the best among all models (AUC = 0.789, Figure 2). NRI analysis showed that compared to the AHA lesion type model (type IV–V, VI, VII, VIII) the accuracy of the combined models (AHA lesion type IV–V + stenosis degree + TBR, AHA lesion type VI + stenosis degree + TBR, AHA lesion type VII + stenosis degree + TBR, AHA lesion type VIII + stenosis degree + TBR) for predicting symptomatic plaques improved by 44.5%, 13.9%, 50.7%, 38.3%, respectively (Figure 3 and Figure 4, Supplementary Materials Table S2).

## 4. Discussion

In this study, we evaluated the morphological, composition and metabolic features of 402 advanced carotid plaques in 280 patients using integrated PET/MRI. The prevalence of AHA lesion type IV-V and type VII in symptomatic plaques varied under different inflammatory uptake status. Compared with AHA lesion-types alone, PET uptake and stenosis degree could significantly improve the classification of vulnerable plaques, especially for AHA lesion type IV-V and type VII plaques.

### 4.1. Association between Morphological and Inflammation Features of Carotid Plaque

PET is the most clinically proven technique to evaluate inflammation, owing to the assistance of high-sensitivity radioactive tracer ^18^F-FDG [21]. The relationship between morphological characteristics and the intensity of ^18^F-FDG uptake has been previously evaluated using PET/CT in both normal appearance carotid artery and advanced carotid plaque. ^18^F-FDG uptake was significant higher in symptomatic plaques compared to that in asymptomatic [22,23,24], which is consistent with the present findings. The degree of luminal stenosis is the only morphological indicator to determine whether patients with advance carotid plaque should undergo revascularization, according to the current guideline from the European Society for Vascular Surgery (ESVS) [25]. Moreover, the risk score including ^18^F-FDG uptake and stenosis severity in carotid plaque proposed by Kelly et al. [13] could be used to improve the identification of recurrent stroke. Previous studies have preliminarily described the relationship between morphology and inflammatory features of carotid plaque. However, histopathologic studies have demonstrated considerable differences in rupture risk between plaques with identical morphological features [26]. Further studies have found that the specific components of the plaque, such as intraplaque hemorrhage, lipid core, and calcification, were the reasons for this difference [7]. The relationship between occurrence of calcification, CT low-density plaque, and ^18^F-FDG uptake have been previously evaluated by PET/CT study [27]. However, it is difficult to further investigate other components such as fibrous, lipid, and hemorrhage of advanced plaque with CT because of overlap in Hounsfield units and the small size of these lesions.

### 4.2. Association between Component and Inflammation Features of Advanced Carotid Plaque

In this study, we took advantage of simultaneous acquisition of ^18^F-FDG PET and MR vessel wall imaging to compare the component and inflammation characteristics of advanced carotid plaques. Owing to its high resolution, MR vessel wall imaging seems particularly well suited for the characterization of vulnerable plaques according to the AHA lesion-types [16]. The relationship between complex compositions of carotid plaques with MRI and the intensity of ^18^F-FDG uptake has been previously evaluated using two separate imaging sessions [28]. High ^18^F-FDG uptake was associated with lipid core [29], intraplaque hemorrhage [30] compared to fibrous tissue and calcification. However, the potential risk of inaccurate uptake measure caused by poor spatial registration between the two separate imaging sessions restricted the use of inflammation evaluation in carotid plaque. Integrated PET/MRI system provided the insight to the precise and simultaneous analysis of ^18^F-FDG PET images of carotid arteries [31]. High prevalence of AHA lesion type VI as well as high ^18^F-FDG uptake was found in symptomatic carotid plaques in the PET/MRI study [32], and this finding was consistent with ours. Though Hyafil [32] et al. failed to find any significant difference for other AHA lesion types between symptomatic and asymptomatic plaques, significantly lower prevalence of AHA lesion type IV-V and type VII were found in the symptomatic plaques in this study. In addition, another interesting phenomenon was observed in this study that AHA lesion type IV-V was less prevalent in the symptomatic arteries (32.2% vs. 46.3%). This unusual finding seems to go against the convention that symptomatic plaques have larger lipid content [33]. However, it is in accordance with previous suggestions by Sadat et al. [34] that at the time of plaque rupture inevitably there was escape of lipid-rich atheromatous debris from the plaque. After the escape of atheromatous debris, plaque may be left only with MR-evident fibrous content. Sadat et al. [34] also found that asymptomatic ruptured plaque had higher percentage lipid volume than asymptomatic non-ruptured plaques (61% vs. 25%). The process that asymptomatic evolved to symptomatic plaque might be accompanied by the dynamic changes of morphologic and component features. Such asymptomatic plaques with large lipid content might have reached a ‘pre-symptomatic’ state at which they are becoming high-risk, hence the early classification as well as further intervention to stop or slow down the process is needed.

### 4.3. Incremental Value of ^18^F-FDG PET in Classification Pre-Symptomatic Stage of Advanced Carotid Plaque 

The potential risk factors which prompt asymptomatic plaque to evolve into pre-symptomatic and ultimately rupture are not fully understood. Recent studies have preliminarily demonstrated several morphological and component features, such as degree of luminal stenosis [4], intraplaque hemorrhage [8], and lipid core [9], which independently predict future cardiovascular events in asymptomatic persons with subclinical plaques. However, the understanding of the role of plaque inflammation in first-ever clinical manifestations of cardiovascular disease in asymptomatic persons with subclinical atherosclerosis remains limited, as was explicitly highlighted by a PET/CT study of symptomatic patients [35]. In the present study, we found the interesting result that the prevalence of AHA lesion types in symptomatic and asymptomatic plaques varies under different inflammatory uptake status, which can be summarized into the following three patterns. (1) The prevalence of AHA lesion type VI in symptomatic plaque was significantly higher than that in the asymptomatic group. This relationship remained unchangeable under both inflammation status, indicating complex plaque with possible surface defect, hemorrhage, or thrombus had a high probability of representing an active state no matter what the inflammation status was. (2) There was no significant difference in the prevalence of AHA lesion type VIII between symptomatic and asymptomatic plaque, and this relationship also remained unchangeable under both inflammation status, indicating fibrotic plaque was in a stable state. (3) The prevalence of AHA lesion type IV-V and type VII were higher in asymptomatic plaque, and the differences were only significant under specific inflammation status, indicating inflammation played an important role in evolution for plaque with a lipid or necrotic core and plaque with calcification. Inflammation features measured by ^18^F-FDG could optimize the AHA lesion-types to identify the more vulnerable, pre-symptomatic stage carotid plaque.

There were several limitations in this study. First, histological validation of AHA lesion-types in the carotid plaques could not be provided. Second, further quantitative evaluation of volume of each plaque component was not performed. Third, the potential value of ^18^F-FDG uptake in stroke risk classification needed to be validate in the longitudinal cohort. Forth, an artificial intelligence and radiomics model should be further developed to predict FDG uptake status of carotid plaques and offer a feasible method for optimizing PET examinations to evaluate the risk of stroke.

## 5. Conclusions

In summary, integrated PET/MRI could simultaneously evaluate the morphological component and inflammation features of advanced atherosclerotic plaques and provide supplementary optimization information over AHA lesion-types for identify vulnerable plaques in atherosclerosis subjects. Longitudinal cohort is needed to further validate the incremental value of PET/MRI in future stroke risk prediction.

## Figures and Tables

**Table 1:** Patient population.

	*n*	%
Vascular risk factors/comorbidities		
Active smoking	92	32.9
Former smoking	54	19.3
Hypertension	213	76.1
Diabetes mellitus	109	38.9
Hyperlipidemia	90	32.1
Coronary artery disease	68	24.3
Overweight (body mass index > 25 kg/m^2^)	155	55.4
Baseline medication		
Antiplatelet agent plus statin	188	67.1
Statin only	13	4.6
Antiplatelet agent only	10	3.6
Neither antiplatelet agent nor statin	69	24.7

**Table 2:** PET/MRI features of symptomatic and asymptomatic advanced plaque.

Features	Symptomatic Plaque (*n* = 87)	Asymptomatic Plaque (*n* = 315)	*p* Value
MRI			
Mean luminal diameter, mm, median (IQR)	2.43 (1.69–2.95)	3.11 (2.32–4.10)	<0.001
Mean wall diameter, mm, $\bar{x}$ ± SD	8.46 ± 1.99	8.39 ± 1.61	0.938
Mean wall thickness, mm, median (IQR)	2.98 (2.40–3.55)	2.43 (1.95–3.04)	<0.001
Stenosis degree, %, median (IQR)	61.5% (53.3–70.5%)	50.0% (38.0–63.0%)	<0.001
Wall area, mm^2^, median (IQR)	55.46 (39.23–75.95)	49.11 (38.47–63.17)	0.069
Luminal area, mm^2^, median (IQR)	4.97 (2.48–7.28)	8.09 (4.57–13.82)	<0.001
Normalized Wall Index, median (IQR)	0.93 (0.88–0.97)	0.85 (0.75–0.93)	<0.001
Total vessel area, mm^2^, median (IQR)	59.05 (43.82–83.22)	60.94 (46.31–76.10)	0.789
Remodeling index, median (IQR)	1.44 (1.06–2.14)	1.36 (1.00–1.77)	0.121
AHA lesion types			
Type IV-V, *n* (%)	28 (32.2%)	146 (46.3%)	0.018
Type VI, *n* (%)	44 (50.6%)	69 (21.9%)	<0.001
Type VII, *n* (%)	8 (9.2%)	77 (24.4%)	0.002
Type VIII, *n* (%)	7 (8.0%)	23 (7.3%)	0.815
PET			
SUV_max_, median (IQR)	2.30 (1.68–2.92)	1.93 (1.52–2.49)	0.007
TBR, median (IQR)	2.96 (2.35–3.80)	2.32 (1.81–3.00)	<0.001

**Table 3:** Multivariate logistic regression analysis and ROC of single component model.

	OR	95% CI		*p* Value	AUC	*p* Value
Stenosis degree	1.742	1.191	2.549	0.004	0.702	<0.001
Type IV–V	5.269	1.178	23.568	0.030	0.485	0.648
Type VI	14.596	3.218	66.202	<0.001	0.666	<0.001
Type VII	4.195	0.852	20.642	0.078	0.454	0.150
Type VIII	7.297	1.329	40.068	0.022	0.512	0.722
SUV_max_	0.947	0.625	1.434	0.799	-	-
TBR	1.456	1.077	1.968	0.014	0.673	<0.001

## Data Availability

The datasets used and/or analysed during the current study are available from the corresponding author on reasonable request.

## Supplementary Materials

The following supporting information can be downloaded at: https://www.mdpi.com/article/10.3390/diagnostics14101006/s1, Table S1. Integrated PET/MR acquisition parameters. Table S2. Prevalence of high and low risk plaque identified by single component model and combined model.
