# Supplementary material for: In Vivo Classification and Characterization of Carotid Atherosclerotic Lesions with Integrated 18F-FDG PET/MRI
Source: Diagnostics (Basel). 2024 May 13;14(10):1006. doi: 10.3390/diagnostics14101006 (PMC11120206; doi:10.3390/diagnostics14101006)
Supplement: Supplementary file 1 [file diagnostics-14-01006-s001.zip › diagnostics-2952396-supplementary.pdf]

Table S1. Integrated PET/MR acquisition parameters

MR sequence	TR/TE (ms)	FOV (mm)	resolution (mm)	slice number	Scan time
**TOF-MRA**	17.5/ 4	180×180	0.75*0.60*1.5	323	5:45
**3D-T1 FSE**	800 / 14.52	180×180	0.60*0.60*0.60	390	6:50
**3D-T2 FSE**	2000 / 198.44	180×180	0.60*0.60*0.60	390	6:36
**3D-T1 FSE C+**	800 / 14.52	180×180	0.60*0.60*0.60	390	6:50

Table S2. Prevalence of high and low risk plaque identified by single component model and combined model

Classification according to single component model (MR AHA type IV-V)	Classification according to combined model (MR AHA type + stenosis degree + TBR)		
	high risk	low risk	total
High risk (Type IV-V)			
Symptomatic	23	5	28
Asymptomatic	72	74	146
NRI, %			
Symptomatic	-17.9		
Asymptomatic	50.7		
Overall	32.8		
Low risk (other types)			
Symptomatic	56	5	61
Asymptomatic	128	149	277
NRI, %			
Symptomatic	91.8		
Asymptomatic	-46.2		
Overall	45.6		
Total			
Symptomatic	79	10	89
Asymptomatic	200	223	423
NRI, %			
Symptomatic	57.3		
Asymptomatic	-12.8		
Overall	44.5		

Classification according to single component model (MR AHA type VI)	Classification according to combined model (MR AHA type + stenosis degree + TBR)		
	high risk	low risk	total
High risk (Type VI)			
Symptomatic	44	0	44
Asymptomatic	67	2	69
NRI, %			
Symptomatic	0		
Asymptomatic	2.9		
Overall	2.9		
Low risk (other types)			
Symptomatic	29	16	45
Asymptomatic	81	273	354
NRI, %			
Symptomatic	64.4		
Asymptomatic	-22.9		

Overall	41.6		
Total			
Symptomatic	73	16	89
Asymptomatic	148	275	423
NRI, %			
Symptomatic	32.6		
Asymptomatic	-18.7		
Overall	13.9		

Classification according to single component model (MR AHA type VII)	Classification according to combined model (MR AHA type + stenosis degree + TBR)		
	high risk	low risk	total
High risk (Type VII)			
Symptomatic	3	5	8
Asymptomatic	15	62	77
NRI, %			
Symptomatic	-62.5		
Asymptomatic	80.5		
Overall	18.0		
Low risk (other types)			
Symptomatic	72	9	81
Asymptomatic	166	180	346
NRI, %			
Symptomatic	88.9		
Asymptomatic	-48.0		
Overall	40.9		
Total			
Symptomatic	75	14	89
Asymptomatic	181	242	423
NRI, %			
Symptomatic	75.3		
Asymptomatic	-24.6		
Overall	50.7		

Classification according to single component model (MR AHA type VIII)	Classification according to combined model (MR AHA type + stenosis degree + TBR)		
	high risk	low risk	total
High risk (Type VIII)			
Symptomatic	7	0	7
Asymptomatic	22	1	23
NRI, %			
Symptomatic	0		

Asymptomatic	4.3		
Overall	4.3		
Low risk (other types)			
Symptomatic	73	9	82
Asymptomatic	186	214	400
NRI, %			
Symptomatic	89.0		
Asymptomatic	-46.5		
Overall	42.5		
Total			
Symptomatic	80	9	89
Asymptomatic	208	215	423
NRI, %			
Symptomatic	82.0		
Asymptomatic	-43.7		
Overall	38.3		
